# Competition, Nodule Occupancy, and Persistence of Inoculant Strains: Key Factors in the *Rhizobium*-Legume Symbioses

**DOI:** 10.3389/fpls.2021.690567

**Published:** 2021-08-19

**Authors:** Marcela Mendoza-Suárez, Stig U. Andersen, Philip S. Poole, Carmen Sánchez-Cañizares

**Affiliations:** ^1^Department of Molecular Biology and Genetics, Aarhus University, Aarhus, Denmark; ^2^Department of Plant Sciences, University of Oxford, Oxford, United Kingdom

**Keywords:** bioinoculants, biofertilizers, competition, rhizobia, sustainable agriculture, symbiosis, biological nitrogen fixation, legume

## Abstract

Biological nitrogen fixation by *Rhizobium*-legume symbioses represents an environmentally friendly and inexpensive alternative to the use of chemical nitrogen fertilizers in legume crops. Rhizobial inoculants, applied frequently as biofertilizers, play an important role in sustainable agriculture. However, inoculants often fail to compete for nodule occupancy against native rhizobia with inferior nitrogen-fixing abilities, resulting in low yields. Strains with excellent performance under controlled conditions are typically selected as inoculants, but the rates of nodule occupancy compared to native strains are rarely investigated. Lack of persistence in the field after agricultural cycles, usually due to the transfer of symbiotic genes from the inoculant strain to naturalized populations, also limits the suitability of commercial inoculants. When rhizobial inoculants are based on native strains with a high nitrogen fixation ability, they often have superior performance in the field due to their genetic adaptations to the local environment. Therefore, knowledge from laboratory studies assessing competition and understanding how diverse strains of rhizobia behave, together with assays done under field conditions, may allow us to exploit the effectiveness of native populations selected as elite strains and to breed specific host cultivar-rhizobial strain combinations. Here, we review current knowledge at the molecular level on competition for nodulation and the advances in molecular tools for assessing competitiveness. We then describe ongoing approaches for inoculant development based on native strains and emphasize future perspectives and applications using a multidisciplinary approach to ensure optimal performance of both symbiotic partners.

## Introduction

Biological nitrogen fixation (BNF) is an important source of nitrogen, and the various legume crops and pasture species often fix as much as 200 to 300 kg of nitrogen per hectare per year (Peoples et al., 1995). Altogether, the legume-*Rhizobium* symbioses contribute the equivalent of approximately a quarter of the nitrogen applied to arable land annually as chemical fertilizers (Herridge et al., 2008). The use of legumes in rotations also offers control of crop diseases and pests (Graham and Vance, 2000). The benefits of the symbioses between legumes and nitrogen-fixing bacteria are crucial in farming systems worldwide. Rhizobia are ubiquitous in soil but show great variation in their number and composition of natural populations depending on properties of the soils (Brockwell et al., 1995; Hirsch, 1996; Vuong et al., 2017). Their ability to form nodules in the presence of other strains determines their nodulation competitiveness (referred to here as rhizobial competitiveness; Yates et al., 2011; Onishchuk et al., 2017). While nitrogen fixation in an inoculated pasture is assumed to be due to the strain used in the inoculant, the identity of the strains occupying the nodules is generally unknown (Irisarri et al., 2019). Several rhizobia strains can inhabit nodules within the same host plant, even co-inhabiting the same nodule (Mendoza-Suárez et al., 2020), and compete for host resources with non-fixing (“cheating”) strains (Checcucci et al., 2017). However, it has been shown that plants can sanction nodules that are inefficient at fixing nitrogen (Kiers et al., 2003; Regus et al., 2017; Westhoek et al., 2021). Moreover, legumes are able to control the number of nodules formed through an “autoregulation” process in which a shoot-derived signal limits infection (Kinkema et al., 2006; Ferguson et al., 2010; Reid et al., 2011; Mortier et al., 2012). The phytohormone ethylene also has an inhibitory effect on nodulation in most legumes (Penmetsa and Cook, 1997; Penmetsa et al., 2003; Lin et al., 2020), although rhizobia can influence these mechanisms by altering ethylene levels via the production of a rhizobiotoxine which inhibits ethylene biosynthesis of host roots (Ma et al., 2002; Sugawara et al., 2006).

Rhizobial competitiveness has important practical implications for agriculture, as differences in nitrogen fixation efficiency between strains can be large (Slattery and Pearce, 2002; Irisarri et al., 2019). Elite rhizobial inoculants must be highly effective in providing the plant with fixed nitrogen (N_2_-effectiveness) and, at the same time, be highly competitive for nodule occupancy (competitiveness) in a background of native rhizobia that may show high competitiveness combined with low N_2_-effectiveness (Checcucci et al., 2017; Onishchuk et al., 2017).

Microbial interactions in agriculture are part of a multicomponent equation that includes (i) plant genotype, (ii) environment, and (iii) plant and soil microbiomes. These factors should be taken into account when evaluating the success of beneficial microbes (Sessitsch et al., 2002; Busby et al., 2017; Onishchuk et al., 2017; diCenzo et al., 2019). Nodule formation, and therefore rhizobial competitiveness, is affected by soil type and its physicochemical properties (i.e., temperature, pH, and moisture; Hungria and Franco, 1993; Frey and Blum, 1994; Anyango et al., 1995; Montañez et al., 1995; Zahran, 1999; Rao et al., 2002; Rathi et al., 2018), nutrient availability and the ability of microbes to use them (Rynne et al., 1994; Kyei-Boahen et al., 2017; Kasper et al., 2019), the population of native rhizobia and the remaining soil microbiome (Meade et al., 1985; Siefert et al., 2018; Han et al., 2020), previous inoculation history (Laguerre et al., 2003; Batista et al., 2015), and/or the choice of inoculation method (Danso and Bowen, 1989; López-García et al., 2009). The degree to which the rhizobial strains adapt to the local soil conditions will strongly influence the competition between strains.

**FIGURE 1:**
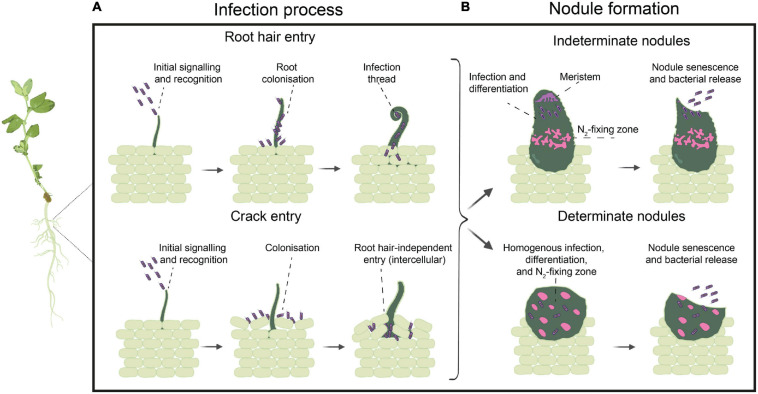
Infection process and nodule formation. **(A)** Infection process steps taking place in the case of root hair entry, with root colonization followed by root hair curling and intracellular invasion by the formation of infection threads (ITs), or crack entry, with colonization occurring between root epidermal cells followed by intercellular root cortical cells invasion of free-living cells (in purple). **(B)** Nodule formation and bacteroid differentiation (larger, pink cells), with a defined spatial distribution in the case of indeterminate nodules, harboring undifferentiated cells in the nodule meristem and differentiated and branched bacteroids in the nitrogen-fixing zone, versus and homogenous infection zone in the determinate nodules, harboring both non-differentiated cells and rounded and differentiated bacteroids together. Once the nodule senesces, the bacterial cells are released back into the soil.

But how is this endosymbiotic relationship with legumes established? Nitrogen-fixing rhizobia have complex life cycles (Poole et al., 2018). Rhizobia are found as free-living bacteria in soil and in the rhizosphere, which are highly heterogeneous environments in both space and time. In brief, the symbiosis is initiated in the rhizosphere following an exchange of signals between both partners (Oldroyd and Downie, 2004; Oldroyd et al., 2011; Udvardi and Poole, 2013; Figure 1A). Root exudates released into the rhizosphere are also part of this chemical dialogue (Badri and Vivanco, 2009), being specific for the plant genotype (Monchgesang et al., 2016) and changing during the life cycle of the plant, the root zone, the rhizosphere microbial community and with time (Zhalnina et al., 2018; Canarini et al., 2019; Korenblum et al., 2020). In nature, host legumes are surrounded by other plants, and other roots or mesofauna may also exude chemoattractants, adding noise to the host root exudate (De-la-Peña et al., 2008). Therefore, bacterial attraction and initial root colonization occurs at multiple stages that are defined by spatial and temporal separation (Massalha et al., 2017; Poole, 2017). Indeed, a microfluidics-based approach combined with advanced microscopy showed that the first step in colonization seems to involve newly divided and undifferentiated cells in the nutrient-rich microenvironment of the root elongation zone (Massalha et al., 2017). Bacterial accumulation at this site occurs prior to the attachment to differentiated root-hair cells (Massalha et al., 2017; Poole, 2017). They usually adhere to roots using adsorption, root attachment being affected by soil and root physicochemical properties, such as pH, Ca^2+^ and Mg^2+^ concentrations and water availability (Rodríguez-Navarro et al., 2007).

The infection process starts when rhizobia enter root systems through natural cracks between epidermal cells at the base of emerging lateral roots (termed “crack entry”), or, more commonly, when compatible rhizobia induce curling and deformation of growing root hairs around the bacterial cells that subsequently enter through an intracellular infection thread (IT, Figure 1A; Rae et al., 2021). Crack entry infection is considered more primitive than ITs because the host does not experience sophisticated cellular differentiation of root hairs (Sprent, 2008; Gage, 2019), although some species of plants (e.g., *Lotus japonicus*) can alter their mode of infection depending on the site of infection (Montiel et al., 2021). Only the emerging root hairs are infectable, with polar root hair growth required to achieve the necessary root hair deformation and cell wall invagination to form an IT (Turgeon and Bauer, 1985; Esseling et al., 2003). Hence, this process is a transient phenomenon, as root hairs remain infectable for only a few hours in a given root region. Whereas the transiency of root hair infectability highlights the relevance of the reversible and generally polar attachment to emerging root hairs, the irreversible attachment and biofilm formation seems relevant for rhizosphere and soil colonization, although not for infection and nodulation, as it would be completed only after the root hairs are no longer infectable. IT initiation and elongation continues through cortical cell layers until rhizobia are released into dividing cortical cells. They are taken up into the plant cytosol and surrounded by a plant-derived membrane (Figure 1B). The rhizobial cells with their plant surrounding membrane are known as symbiosomes, which are temporary plant organelles where nitrogen fixation takes place (Gage, 2019; Pérez-Giménez et al., 2021). In the symbiosomes, rhizobia adjust their metabolism in response to the stress conditions, differentiating into bacteroids, after which the nitrogenase complex is expressed, leading to active N_2_-fixation (Poole et al., 2018; Ledermann et al., 2021). There is no general consensus on the morphological types of legume nodules and their internal structure. Although some authors use a classification system with up to five nodule types – aeschynomenoid, desmodioid, indeterminate, lupinoid, and primitive (Sprent, 2008) – the major types of nodule shape and structure are determinate and indeterminate nodules (Figure 1B). Determinate nodules are spherical, lacking a persistent meristem and therefore cease growing at some point, whereas indeterminate nodules are elongated, with a persistent meristem, resulting in indefinite growth and branching in irregular shapes (Ferguson et al., 2010). These two patterns of nodule development differ also in bacteroid differentiation, with determinate nodules showing a more synchronic, time-dependent and homogeneous maturation of most rhizobia within the nodules, in contrast to indeterminate nodules, in which rhizobia at different stages of differentiation are observed within the same nodule, with bacteroid maturation depending on the different nodule spatial zones (Ferguson et al., 2010; Poole et al., 2018). Bacteroids in determinate nodules are viable and retain their morphology and their capacity to divide and revert to free-living cells, whereas in indeterminate nodules, only a small fraction of rhizobial cells in the saprophytic zone of nodules remains in a viable and vegetative form, being able to multiply in the infection threads and regrow outside the nodule, increasing the bacterial population in the soil after nodule senescence (Mergaert et al., 2006; Montiel et al., 2017). When the nodule senesces, the saprophytic zone that is formed is where rhizobia are nourished by the products of organic breakdown and decay and multiply massively (Timmers et al., 2000; Wielbo et al., 2010). What we do not yet understand is which phases in the nodulation process are exposed to competition.

The improvement of symbiotic nitrogen fixation is one of the main challenges facing agricultural research. It is therefore necessary to evaluate nodule occupancy in order to assess the competitiveness of a strain. Although competition assays must include field assessments that confirm the results obtained from experiments under controlled conditions, there are limitations when they are performed at a large scale. Here, we will review the molecular factors that can influence rhizobial competitiveness and how the development of molecular tools is simplifying this task and we will discuss the implications of rhizobial competitiveness for the establishment of a successful symbiosis and for the search for elite rhizobial strains. We will also emphasize the importance of performing N_2_-effectiveness and competitiveness assays using relevant field soils and plant genotypes. Although crop management and environmental factors (for example: soil pH, soil temperature, salinity, moisture, soil texture, among others) are not discussed in this review, these factors can also influence rhizobial competitiveness and, therefore, future inoculant performance (reviewed in detail by Vlassak and Vanderleyden, 1997; Saad et al., 2020).

## Types of Competition and Genetic Features Influencing Competitiveness

Competition for nodulation is a key adaptive feature of rhizobia that is of great importance in the practical application of inoculants. However, the genetic basis of competitiveness for nodule formation is not fully understood yet. What we know is that competitiveness, similarly to nitrogen fixation, is controlled by specific genes that are expressed during different time points in nodule development (Ampe et al., 2003; Barnett et al., 2004). In addition, rhizobia are part of the plant microbiome community and, as such, they have to interact with the non-rhizobial accompanying members of this dynamic community in order to undertake a range of beneficial functions including growth promotion, nutrient acquisition, pathogen resistance or stress tolerance, thus, exploiting the versatile benefits offered by plant-microbe interactions (as reviewed by Compant et al., 2019; Trivedi et al., 2020). The assemblage of the plant microbiome community is currently the focus of active investigation, as different components have been shown to have a crucial impact in the assembly process, such as the soil and seed-born initial microbial repertoires, both accounting for key microbe-microbe interactions within this microbial consortium (Philippot et al., 2013; Turner et al., 2013; Zgadzaj et al., 2016; Nelson, 2018). Indeed, Plant Growth-Promoting Rhizobacteria (PGPR) have been found to be enriched in the soybean rhizosphere, reinforcing the idea that rhizosphere recruitment is an important first step in symbiotic interactions (Liu et al., 2019). Hence, elite inoculant behaviors appear to be a consequence of gene assortment, with genes involved in an efficient symbiosis with their plant hosts, PGPR traits, or secretion systems (Pastor-Bueis et al., 2019). Onishchuk et al. (2017) identified two types of competition influenced by different factors: exploitative (indirect) competition, involving a more effective use of a common limiting nutrient, or interference (direct) competition, whereby other cells are prevented from growing and surviving in the environment. Hence, to be both competitive and N_2_-effective, an elite strain should have genetic components that enable it to (i) successfully colonize the rhizosphere and benefit from available nutrients in an effective way, (ii) prevent the growth of other bacterial cells, (iii) establish an efficient symbiosis, and (iv) promote plant growth. Here, we will review some of the components that are relevant to the four points mentioned above and summarized in Table 1. The genetic knowledge on the pre-infection, infection and nodulation stages has mainly been gained from experiments with defective mutants and, more recently, with high-throughput technologies. Hence, although these data come from laboratory experiments, such traits may also be present in the soil rhizobia populations. Manipulating the expression of these traits, either genetically or through culture conditions, may lead to improvements in the ability of rhizobial strains to compete against endogenous soil populations.

**TABLE 1:** Examples of some of the genes reported to be involved in competition.

**Category**	**Gene function**	**Gene name**	**Rhizobial strain object of the study**	**Publication**
*(i) Genetic Components for Rhizosphere Colonization*	Motility	*motA, motB flg* and *fli* genes	*E. meliloti* L5-30	Bauer and Caetano-Anollés, 1988
	Chemotaxis	*cheAWRBYD* (Che1 cluster)	*R. leguminosarum* bv. *viciae* 3841	Miller et al., 2007; Wheatley et al., 2020*
	ABC transporters	*teuBAC1C2 (root exudates) aapJQMP (branched-chain amino acids) livM (branched-chain amino acids)*	*R. tropici CFN299 R. leguminosarum E. meliloti* 2011	[Bibr B252][Bibr B104][Bibr B231]*
	EPS biosynthesis	*dgoK pssA*	*E. meliloti R. leguminosarum* bv. *trifolii*	Geddes et al., 2014 Janczarek et al., 2009
		*pssD*	*R. leguminosarum* bv. *viciae* 3841	Wheatley et al., 2020*
		*exoY*	*S. meliloti* 1021	Jones, 2012
	Lypopolysaccharide biosynthesis	*lpsB acpXL*	*E. meliloti* 2011/1021	Niehaus et al., 1998; Lagares et al., 1992; Campbell et al., 2002; Sharypova et al., 2003
	Attachment	*praR*	*R. leguminosarum* bv. *viciae* 3841	Frederix et al., 2014
	Rhizopine biosynthesis and catabolism	*mosABC mocCABRDEF*	*E. meliloti* L5-30	Gordon et al., 1996; Murphy et al., 1987
	PHB synthesis and degradation	*phaC bdhA*	*E. meliloti*	Aneja et al., 2005
	Homoserine catabolism	pRL80079-pRL80088	*R. leguminosarum* bv. *viciae* 3841	Vanderlinde et al., 2014
	Proline catabolism	*proDH*	*E. meliloti* GRM8	Jimenez-Zurdo et al., 1995
	Rhamnose catabolism	*rhaRSTPQUK, rhaDI*	*R. leguminosarum* bv. *trifolii* Rlt100	Oresnik et al., 1998
	Myo-inositol catabolism	*iolDEB iolA, iolRCDEB*	*R. leguminosarum E. meliloti* 2011	[Bibr B88][Bibr B231]*; Kohler et al., 2010
	Glycerol catabolism	*glpDSTPQUVK*	*R. leguminosarum* bv. *viciae* VF39	Ding et al., 2012
	Transcriptional regulation	*rsh rosR*	*B. diazoefficiens* USDA 110 *R. etli* CE3 *R. leguminosarum bv. trifolii* 24.2	[Bibr B225][Bibr B33][Bibr B127]; Rachwał et al., 2016
*(ii) Genetic components to prevent the growth of other bacterial cells*	Bacteroicin production	*cinRIS* (small bacteriocin) *tfxABCDEFG* (trifolitoxin)	*R. leguminosarum Rhizobium trifolii* T24	[Bibr B266][Bibr B268]; Triplett and Barta, 1987
	T1SSd (biofilm production)	*prsD* and *prsE*	*R. leguminosarum* bv. *viciae*	Russo et al., 2006
	Type III secretion system (T3SS)	*rhcJ, rhcLNQRSTU, hrpW, rhcVD, nops* genes *nopP*	*S. meliloti Rhizobium* sp. NGR234	Jimenez-Zurdo et al., 1995 Marie et al., 2003 Ausmees et al., 2004
	Type IV secretion system – pilus (T4SS)	*virB1-virB11*	*S. meliloti*	Nelson et al., 2017
	Type VI secretion system (T6SS)	*tssHD tssABC1C2 tagH tssEFGGHKLMFE*	*R. etli* Mim1	Salinero-Lanzarote et al., 2019
*(iii) Genetic components to establish an efficient symbiosis*	Nodulation	*nodD*	*R. tropici* strain CIAT 899 *R. leguminosarum* bv. trifolii	del Cerro et al., 2015 Ferguson et al., 2020
*(iv) Genetic components to promote plant growth*	Tryptophan biosynthesis (precursor of IAA and auxin)	*trpEF trpC trpF*	*E. meliloti* 2011	[Bibr B22][Bibr B231]*
		*trpB*	*R. etli CE3*	Taté et al., 1999
	Phosphate solubilization	*phoR, phoUB ptsSCAB*	*E. meliloti* 2011	Pobigaylo et al., 2008*
	Siderophore production (Rhizobactin 1021)	*rhbABCDEF rhtA, rhrA*	*E. meliloti* 1021	Lynch et al., 2001

*Studies with several other genes reported to affect competition, but not all included in this table.

### Genetic Components for Rhizosphere Colonization

When it comes to exploitative competition, where bacteria compete for the same common resources without directly interacting, bacterial chemotaxis toward exuded compounds is an important trait for root colonization and plant-driven selection of microorganisms (Bais et al., 2006; Raina et al., 2019). Motility and chemotaxis are factors affecting nodulation efficiency and competitiveness (Mellor et al., 1987; Caetano-Anollés et al., 1988; Bauer and Caetano-Anollés, 1990). Disruption of the flagellum hook gene *flgE* in *Mesorhizobium tianshanense* caused a flagellar-less phenotype, leading to the complete loss of swimming ability, a heavier biofilm formation and decreased bacterial attachment on the root hair. These *in vitro* assays suggest that flagella are involved in the early stage of the symbiosis process (Zheng et al., 2015). The major chemotaxis gene cluster of *Rhizobium leguminosarum* bv. *viciae*, Che1, has been shown to be essential for competitive nodulation (Miller et al., 2007; Wheatley et al., 2020). It has to be noted that these observations have been mainly made from laboratory experiments carried out either in liquid media or in flooded substrates and cannot be extrapolated to soil conditions (Iturralde et al., 2019). Motility of inoculated rhizobia in soils at field-capacity is generally scarce; therefore, the distribution of the rhizobia in the soil profile is important, as it facilitates entry of growing roots in contact with the static rhizobia (López-García et al., 2002, 2009). Motility and chemotaxis might only have an effect during the short periods of rainfall or watering, during which the soil pores are water-saturated. Consequently, inoculant competitiveness may be improved through the application of liquid in-furrow inoculants, contributing to rhizobial dispersion, instead of seed-coated dried inoculation.

In the case of root colonization patterns, PGPR share common mechanisms with rhizobia for colonizing roots (Drogue et al., 2012). Attachment of bacteria to root surfaces is a multi-step process. An initial reversible attachment is followed by an irreversible attachment that occurs several hours after initial attachment, and, finally, biofilms can form over a few days (Dazzo et al., 1984; Smit et al., 1992). Two adhesion mechanisms have been described in *R. leguminosarum* to mediate the reversible and polar attachment to root hairs depending on the soil pH: in acidic conditions, rhizobial surface polysaccharide glucomannan binds to plant lectin, expressed on root-hair tips (Laus et al., 2006; Williams et al., 2008). Whereas under neutral or alkaline conditions, root lectins are solubilised and rhicadhesin, a hitherto unidentified calcium-binding protein, was proposed long ago to facilitate attachment to root hairs (Smit et al., 1989), although the gene encoding it is still a mystery. When competing with the wild type during nodule infection, the glucomannan mutant (*gmsA*) was strongly outcompeted (Williams et al., 2008). There are also other plant components influencing the attachment of *R. leguminosarum* to surfaces such as an arabinogalactan protein (Xie et al., 2012). Weak adherence and reversible attachment are mediated mainly by proteins and anchoring, while stronger adherence and irreversible attachment is mediated by polysaccharides (reviewed by Wheatley and Poole, 2018). Rhizobial adhering proteins (Raps) promote attachment and aggregation by rhizobia (Ausmees et al., 2001). Mutation of the transcriptional regulator *praR*, modulating the expression of the genes encoding Raps, results in enhanced *in vitro* biofilm formation, attachment to root hairs and increased nodulation competitiveness primarily due to the enhanced expression of Rap proteins (Frederix et al., 2014). Cyclic glucans (CG), exopolysaccharides (EPS), lipopolysaccharides (LPS), and capsular polysaccharides (KPS) are the main rhizobial surface polymers required for successful nodulation (Margaret et al., 2011). EPS is an extracellular carbon polymer weakly associated with the bacterial surface and thus abundantly released into the surrounding environment (Marczak et al., 2017). The recognition of EPS by specific LysM receptors modulates plant-bacteria recognition and potentially competition for nodulation (Geddes et al., 2014; Kawaharada et al., 2015). The two basic nodule morphologies appear to exhibit different rhizobial exopolysaccharide requirements. In rhizobia inducing determinate nodules, EPS mutants still induce effective nodules, with EPS playing a signaling role at the late stages of both infection thread initiation and bacterial release (Kelly et al., 2013). However, in symbioses forming indeterminate nodules, EPS is absolutely essential for a successful interaction (reviewed by Fraysse et al., 2003; Acosta-Jurado et al., 2021). EPS-altered mutants (exhibiting conserved LPS) generated in an isogenic strain, *R. loti* PN184, able to nodulate *Lotus pedunculatus* (determinate nodules) and *Leucaena leucocephala* (indeterminate nodules) showed that they were fully effective on a determinate-nodulating host but ineffective on the indeterminate one (Hotter and Scott, 1991). Working on *L. japonicus*, Kawarada and co-workers showed that perception of EPS synthesized by *M. loti* is important for maintaining an intracellular infection mode, with the plant LysM receptor protein EPR3 acting in the root cortex and nodule primordia to support and sustain the containment of rhizobia and to facilitate an efficient infection process. They observed a reduced and delayed nodulation either in the plant *epr3* mutants or in *M. loti* mutants affected in EPS biosynthesis due to a reduction of the normal intracellular infection thread mode and increased intercellular infection (Kawaharada et al., 2017). Interestingly, ITs of determinate nodules are narrower than those from indeterminate ones, where EPS is a critical component of the internal matrix and relevant for the cortex rhizobial invasion (Stacey et al., 1991). In indeterminate nodules, bacteria have to spread out by means of continuous IT penetration in the new cortex cells, whereas in the determinate nodules they spread by division of already infected cells (Fraysse et al., 2003). Consequently, EPS plays an important role in determining symbiotic competence; indeed, *R. leguminosarum* bv. *trifolii* strains overexpressing the biosynthesis gene *pssA* overproduced EPS and showed enhanced competitiveness, nodule occupancy, and symbiotic effectiveness with *Trifolium pratense* (red clover) in relation to their wild type strains (Janczarek et al., 2009). Likewise, *S. meliloti* 1021 mutant overexpressing the *exoY* gene, which encodes the enzyme responsible for the first step in succinoglycan (EPSI) biosynthesis, resulted in an increased production of this surface polymer and, as a consequence, enhanced symbiotic effectiveness with *Medicago truncatula* plants (Jones, 2012). Succinoglycan is essential for infection thread formation on plant hosts and has been shown to be more important than Nod factors for bacterial survival inside nodules (Maillet et al., 2020). Among other surface polysaccharides, EPS also seems to play an essential role also in protection against host plant defense during early and late symbiotic stages of rhizobial growth, when rhizobia are subjected to a prolonged oxidative burst from their plant hosts (Santos et al., 2001; D’Haeze and Holsters, 2004; Davies and Walker, 2007). Succinoglycan also protects *S. meliloti* against the antimicrobial activity of plant-derived nodule-specific cysteine-rich peptides in nodule occupancy and bacteroid differentiation and potentially against pH stress inside symbiosomes (Montiel et al., 2017; Arnold et al., 2018). Regarding LPS, contrary to what happens with EPS, an intact polymer seems to be needed for the formation of narrow ITs from determinate nodules (Stacey et al., 1991). A *B. japonicum* mutant defective in LPS synthesis was able to attach and induce root hair curling but failed to penetrate the root and induce ITs and nodules in soybean. However, in indeterminate nodules, mutants with alterations in this surface polymer formed ITs, although they led to an altered symbiotic phenotype in *Ensifer meliloti* during the association with alfalfa that affects the timing of nodule emergence, the infection development, and the strain competitiveness for nodulation (Lagares et al., 1992; Campbell et al., 2002; Sharypova et al., 2003). *lpsB* is a nice example of a surface mutation which does not affect the rate of infection initiation (i.e., no change in root nodule distribution) but the rate of nodule development and emergence. Such post-infection delay strongly reduces nodulation competitiveness by a factor of nearly 100. Beside this, the *lpsB* mutation shows how surface changes may also modify the host range for N2-fixation (Niehaus et al., 1998).

A common characteristic found in rhizobiales and other soil bacteria is the large number of ATP-binding cassette (ABC) transporters and methyl-accepting chemiotaxis proteins (M), which allows them to thrive in such a heterogenous and changing environment as soil, and to detect a high number of metabolites and influence their motility (Mauchline et al., 2006). ABC transporters, such as the genes *teuBAC1C2*, required for the utilization of root exudates (Rosenblueth et al., 1998) may confer competitive ability (Oger et al., 1997). The genes encoding the amino acid transporter AapJQMP have been shown to be up-regulated in bean and pea bacteroids, indicating the importance of the plant supply for branched-chain amino acids isoleucine and valine (Green et al., 2019). The assimilation of different nutrients and energy also influences competitiveness at different stages, such as during multiplication and survival in bulk soil (Sessitsch et al., 2002) or multiplication from the nodule environment after root senescence thanks to the ability of catabolizing rhizopines, a compound made by bacteroids and subsequently catabolized by free-living cells of the producing strain (Murphy et al., 1987; Gordon et al., 1996). Recent work on genetically engineered *M. truncatula* and alfalfa plants that produce and exude rhizopine into the rhizosphere opened the door for the regulation of the root microbiome (Geddes et al., 2019b). Plant-supplied carbon can be diverted into storage molecules, such as polyhydroxybutyrate (PHB), increasing future survival and reproduction during free-living life-history stages once released into the soil after nodule senescence (Aneja et al., 2005; Ratcliff et al., 2008). Indeed, model prediction shows that the amount of PHB stored per cell could support the survival of active cells for a few days, or over a century for sufficiently dormant cells (Muller and Denison, 2018). Their PHB quantification experiments with *Bradyrhizobium* field isolates done in starvation conditions suggest that PHB is partitioned asymmetrically in dividing cells and that high-PHB isolates used more PHB over the first month, still retaining sufficient PHB for potential long-term survival in a dormant state. Homoserine is another amino acid shown to confer an advantage on those *R. leguminosarum* bv. *viciae* strains whose Sym plasmid harbors the genetic determinants for the catabolism of this plant-associated compound in the rhizosphere of pea roots (Hynes and O’Connell, 1990). The catabolism of other compounds such as rhamnose, proline, myo-inositol, and glycerol has also been reported to influence competitiveness (Jimenez-Zurdo et al., 1995; Oresnik et al., 1998; Fry et al., 2001; Kohler et al., 2010; Ding et al., 2012). Transcriptional regulators involved in metabolism are also important. A *Bradyrhizobium diazoefficiens* mutant with an impaired *rsh* gene (from *relA*-*spoT* homologous), responsible for a pleiotropic adaptation under stressful and starving conditions known as the stringent response, was less competitive than the wild type in occupying soybean nodules (Pérez-Giménez et al., 2021). A functional *rosR* is also important for competitive nodulation. This regulator has pleiotropic effects leading to defective attachment, infection thread formation, and bacteroid differentiation and senescence (Bittinger et al., 1997; Janczarek et al., 2010; Rachwał et al., 2016). As *pssA*, in terms of competitiveness, an increase in the quantity of nodules formed by strains carrying multiple copies of *rosR* was observed in comparison to *R. leguminosarum* bv. *trifolii* wild type strains (Janczarek et al., 2009).

### Genetic Components to Prevent the Growth of Other Bacterial Cells

In terms of interference competition, one strategy is the production of antibacterial compounds, such as bacteriocins, which may play a role in competition for rhizosphere and root colonization (Hirsch et al., 1980; Oresnik et al., 1999; Venter et al., 2001). The quorum sensing system *cinRIS* is responsible for the production of the small bacteriocin, produced by strains of all three biovars of *R. leguminosarum* and inhibiting the growth of several strains of this species (Schripsema et al., 1996). The antibiotic trifolitoxin also improves rhizosphere colonization and increases competitiveness for nodule occupancy of clover (Schwinghamer and Belkengren, 1968; Triplett and Barta, 1987; Robleto et al., 1998). Members of the nodule microbiome of *Medicago sativa* also produce britacidins and tyrocidines (Hansen et al., 2020), supporting the idea that, in addition to nitrogen fixation, legume root nodules are sites of active antimicrobial production, presumably to provide protection from pathogens that might infect these organs and ensure that nitrogen-fixation activity is preserved. The presence of secretion systems in PGPRs and rhizobial strains may play a role in their plant growth-promoting (PGP) functions and also provides means of interference competition against other strains in the rhizosphere (Gupta et al., 2014). For instance, the T1SSd proteins orthologous to the PrsD and PrsE proteins are required for biofilm formation (Russo et al., 2006). The T3SS, T4SS, and T6SS are generally used to inject effector proteins, such as nodulation outer proteins (Nops), directly into eukaryotic host cells or into other bacteria, which can mediate compatibility with the host in rhizobia, modulating partner choice (Jimenez-Zurdo et al., 1995; Marie et al., 2003; Ausmees et al., 2004; Nelson and Sadowsky, 2015). The secretion of Nops through T3SS in the presence of flavonoids is able to induce the transcription of nodulation genes (Jiménez-Guerrero et al., 2017) and might modulate the plant defense response upon infection (Pérez-Giménez et al., 2021). While T3SS orthologs are present in *R. etli* CFN42, *S. fredii* HH103, *B. japonicum* USDA110, and *Rhizobium* sp. NGR234, they are not common in *R. leguminosarum* or *Sinorhizobium* species (Black et al., 2012). The T4SS, encoded by *traGDCAFBHMR*, is involved in conjugal transfer, whereas the T4SS-pili (*virB1-virB11*) system is involved in the colonization of surfaces in gram-negative bacteria (Juhas et al., 2008). *S. meliloti* with a truncated T4SS was less competitive for nodule formation compared to wild type (Nelson et al., 2017). T6SS, encoded by the *imp* (*tss*) and *hcp* clusters, was first identified in the α-rhizobial strain *R. leguminosarum* (Bladergroen et al., 2003) and shown to be important for nodulation of pea plants. Impaired T6SS mutants in *R. etli* Mim1 have been shown to generate small, white nodules in *Phaseolus vulgaris*, although with similar activity (Salinero-Lanzarote et al., 2019). The authors suggested a positive role for T6SS in competition with other soil bacteria, as it was active at a high cell density and in the presence of plant exudates. Many gram-negative bacteria use this secretion system for killing competitors (Schwarz et al., 2010; de Campos et al., 2017; Allsopp et al., 2020) and presumably even as an inter-bacterial communication system mediated by the T6SS–quorum sensing cross-talk (Gallique et al., 2017). These transport secretion systems are often found in the accessory genome of rhizobial strains.

### Genetic Components for Establishing an Efficient Symbiosis

Rhizobial genomes are extremely variable (MacLean et al., 2007), with the secondary replicons generally more genetically diverse between strains than is the primary chromosome (Galardini et al., 2013). Chromids – large replicons carrying essential genes and adopting plasmid-type maintenance and a *repABC* replication system – appear to contain genus-specific genes in *Rhizobium*, *Ensifer*, and *Agrobacterium* (Harrison et al., 2010). When an organism arrives in a new niche, it needs to adapt to the new environment, undergoing genome expansions due to the duplication of existing genes or the acquisition of new ones through horizontal gene transfer in order to acquire new functions that improve the fitness of these strains in the new environment (Martins Dos Santos et al., 2004; Aguilar et al., 2018). This is the case, for example, with *nodD*. NodD regulates the expression of the *nodABCFE* cluster and is therefore involved in Nod factor production. Nod factor decorations are critical for host specificity and their levels are tightly regulated during infection (Krönauer and Radutoiu, 2021). Five *nodD* reiterations were found in *R. tropici* CIAT899, necessary to engage the symbiont in nodulation with different legume plants (del Cerro et al., 2015). They were also present in different nitrogen-fixing rhizobial strains from *P. vulgaris*, suggesting a potential role in host range (Peralta et al., 2016). In *R. leguminosarum* bv. *trifolii*, a second copy, NodD2, enhanced nodule colonization and competitiveness in symbiosis with clover (Ferguson et al., 2020). These accessory *nod* genes may reflect the variations within the interactions among rhizobial and plant species. Indeed, *nodD* has been extensively used for the identification and classification of rhizobial isolates (Boivin et al., 2020; Fields et al., 2021). Sequence heterogeneity within symbiotic plasmids also shows extensive genomic rearrangements, recombination rates, lateral transfer events, and relaxation or intensification of selective pressures (González et al., 2003). Indeed, the diversity of associations between genomic backgrounds and Sym genotypes may be greater in bulk soils than in nodules, as the first symbiotic organs are formed by the most competitive strains’ genotypes for a given host plant (Louvrier et al., 1996; Laguerre et al., 2003) triggering autoregulation of nodulation which start to inhibit further nodulation (Mortier et al., 2012). Legume hosts seem to select differentially within the same soil populations. As an example, pea and faba bean have been reported to select A1 and B1 *nod* groups of *R. leguminosarum* bv. *viciae* as their symbiotic partner to form nodules (Laguerre et al., 2003; Mutch and Young, 2004; Jorrin and Imperial, 2015). When *R. etli nodC* variants from the centers of bean genetic diversification were inoculated on wild and cultivated *P. vulgaris* (common bean), yield was best when plant and bacteria were from the same geographic origin, suggesting mutual symbiotic selectivity and coevolution (Aguilar et al., 2004). During the growth cycle of beans, more competitive rhizobia will return to the soil after nodule senescence and therefore contribute to an increase in their representation in soil, explaining this synergism (Aguilar et al., 2004). The minimal symbiotic genome has been defined recently in *E. meliloti*, allowing gain-of-function approaches that can be used to elucidate genes in the Sym plasmid, as well as those genes from other highly competitive rhizobia that contribute to nodulation competitiveness (Geddes et al., 2021).

### Genetic Components to Promote Plant Growth

The term biofertilizer comprises formulations of different living microbial cells, either a single strain or multiple strains, that promote plant growth by increasing nutrient availability and acquisition (Riaz et al., 2020). Thus, elite rhizobial inoculants, besides the traits involved in competition, should also combine a subset of other important traits, with PGP abilities among them (Vessey, 2003; Lugtenberg and Kamilova, 2009; Jaiswal et al., 2021). These traits enhance yields and cause positive changes in soil structure and microbial community. The production of phytohormones is a major property that has evolved in plant-associated bacteria. It leads to an increased size of plant root system and, subsequently, the exploitation of a larger soil volume, thus improving the mineral and aqueous nutrition available to the plant (Cai et al., 2018). In most bacterial pathways, auxin biosynthesis mainly relies on tryptophan, which acts as a biosynthetic precursor of indole acetic acid (IAA), a common auxin produced by rhizobacteria, and as a signal inducing *ipdC/ppdC* transcription. *E. meliloti trpE* mutants, blocked at the first step in tryptophan biosynthesis, form N_2_-ineffective symbionts (Barsomian et al., 1992). Similarly, Tn5-induced *B. japonicum* tryptophan synthetase mutants lacked the ability to fix nitrogen and were symbiotically defective (Kummer and David Kuykendall, 1989). This deficiency in N_2_-fixation seems to be most likely due to the pleitropic effects of the amino acid auxotrophy, with the host plant unable to supply the nutrients needed for the endosymbiont to establish an effective symbiosis. Interestingly, the tryptophan auxotroph mutant *trpB* of *R. etli* CE3 was unable to produce Nod factors in free-living conditions unless tryptophan was added to the growth medium, rendering nodules ineffective in its symbiotic partner *P. vulgaris.* This indicated that even though this *trpB* mutant was able to induce the development of a nodule primordium, a shortage of aromatic amino acids during nodule invasion strongly altered its ability to subsequently invade the nodule and differentiate into bacteroids (Taté et al., 1999). Some PGPR of the genera *Bacillus, Enterobacter*, or *Pseudomonas* promote plant growth by solubilizing phosphate from the soil. Genes implicated in phosphate solubilization are the sensor kinase *phoR*, and the genes involved in regulation and transport, *phoUB* and *pstSCAB* (Chhabra et al., 2013). Intriguingly, bean nodule bacteroids seem to be phosphate-limited (Green et al., 2019). The secretion of siderophores also promotes plant growth by enhancing iron uptake and utilization (Liu et al., 2017). These molecules have a high affinity for Fe^3+^, scavenging iron from environmental stocks where soil iron is low and reducing it to Fe^2+^ for uptake and utilization (Kramer et al., 2020). As an example, Rhizobactin 1021 is a hydroxymate siderophore produced by the soil bacterium *E. meliloti* 2011 which appears to contribute to the competitive ability of free-living *E. meliloti* in iron-depleted soils (Persmark et al., 1993; Barton et al., 1996; Lynch et al., 2001).

### Future Understanding of Rhizobial Genomes

Understanding the mechanisms behind competitiveness for nodulation may lead to improved inoculation strategies (Bourion et al., 2018). A plethora of molecular mechanisms play a role in competition, concerning both microbe-microbe, and plant-microbe interactions. Sequencing efforts have improved our understanding of rhizobial genomes, with sequence analysis of whole genomes enabling in-depth studies and comparisons of genome structures (Burghardt et al., 2018; Epstein et al., 2018; Cavassim et al., 2020). The high-throughput identification of genes relevant to competitiveness was first approached with signature-tagged mutagenesis (STM) *in planta*, which allowed screening of hundreds of mutants in one passage through the host (Pobigaylo et al., 2008). This study identified 38 mutations in genes that were not previously known to be involved in competitiveness or symbiosis in *E. meliloti*, confirming 23 with attenuated competitiveness phenotypes when they were tested for competition against the wild type. Among them are some of the genetic determinants already mentioned, such as: *trpC* and *trpF*, involved in the conversion of tryptophan to IAA; *pstA* and *pstC*, encoding components of the high-affinity phosphate transporter system PstSCAB; or *iolA*, encoding a methylmalonate-semialdehyde dehydrogenase involved in myo-inositol catabolism (Pobigaylo et al., 2008). STM also allowed the identification of more than a hundred genes in *E. meliloti* associated with rhizosphere colonization of a host (alfalfa) and a non-host plant (pea; Salas et al., 2017), supporting the ancient character of those genes. The high-throughput identification of essential genes in specific conditions is today performed by transposon-insertion sequencing (Tn-Seq or INSeq), a saturation insertion mutagenesis performed in a pool of colonies in a given environment. Each colony contains a single insertion that can be mapped across an entire genome by next-generation sequencing, allowing the determination of gene fitness at genome-scale (Goodman et al., 2011; Perry and Yost, 2014; Chao et al., 2016). This method has been applied in rhizobia, leading to the elucidation of genes involved in competition, such as the ORF SMc00911, conserved and highly expressed in the nodule. This insertion mutant strain strongly outcompeted the *E. meliloti* 1021 wild type strain (Queiroux et al., 2012). More recently, this method has allowed the identification at a genome-wide scale of genes involved in various stages of the rhizobial lifestyle, including those genes required when in competition with other bacteria, which were traditionally missed in the artificial environment of inoculation with a single strain (Wheatley et al., 2020). Among the mutants assesed in this study for their ability to compete against wildtype to form nodules were the genes encoding the chemotaxis protein CheA, glutamine synthetase II (*glnII*), or the polysaccharide biosynthesis protein PssD. A more powerful method moving this field forward are unique but random DNA barcodes used in insertional mutagenesis (Bar-Seq). The abundance of transposon insertions can be followed with a single PCR step to amplify the barcodes followed by NGS, allowing the study of gene fitness across multiple growth conditions (Robinson et al., 2014; Wetmore et al., 2015). Multi-strain barcoding can be used for analyzing how bacteria interact with the plant and one another during competitive root colonization competition coupled to sequencing (Cole et al., 2017; Knights et al., 2021).

Active competition between rhizobial strains might take place throughout the entire course of the symbiosis, from the recruitment of the endosymbionts in the rhizosphere to nodule decay (Wielbo et al., 2010). These methods will improve our understanding of which phases in the nodulation process are exposed to competition. Indeed, although much less explored relative to competition in the rhizosphere, we have previously seen that competition also takes place inside plant nodules, highlighting the complexity of the interactions between the plant host and rhizobia. Studies on rhizobial competitiveness have revealed that competition between strains also extends to the process of infection thread initiation and the growth of rhizobia in the infection threads (Stuurman et al., 2000; Duodu et al., 2009). The INSeq work carried out in *R. leguminosarum* bv. *viciae* has shown that the chemotaxis cluster Che2 is needed in the infection thread (Wheatley et al., 2020). Numerous molecular techniques are available for the evaluation of competitive abilities of rhizobia in the rhizosphere, whereas the formation of infection threads remains poorly understood. New methods based on Periodic Acid-Schiff to visualize the three-dimensional structure of infection threads in sufficient detail using novel and traditional cell wall fluorescent labels combined with laser confocal scanning microscopy presents an exciting opportunity for research in this area, including competition (Rae et al., 2021).

## Methods to Assess Rhizobial Competitiveness for Nodulation

Experiments involving single- and multi-inoculation with pea plants have shown that the nodulation ability of a strain does not predict its competitiveness for nodulation and is not correlated with its N_2_-effectiveness (Bourion et al., 2018). The high competitiveness of a given strain does not ensure high nitrogen-fixing efficiency or high biomass production for the plant, indicating that competitiveness for nodulation is controlled by multiple genetic factors from both the host and the rhizobia strain. This study highlighted that competitiveness for nodulation and nitrogen fixation efficiency must both be considered as selection criteria for improving pea crop production. Although many unknows remain regarding the molecular and genetic mechanisms driving competition for nodulation, it has become clear that, in the development of elite rhizobial inoculants, it is necessary to consider colonization and competition for nodulation separately from symbiotic nitrogen fixation abilities (Checcucci et al., 2017). Therefore, one of the main challenges is still the selection of elite rhizobial strains based on their high performance in the field due to their symbiotic performance, combined with relevant genetic features (Checcucci et al., 2017; Aguilar et al., 2018).

Despite one of the first co-inoculation assays being presented in 1930 by Löhnis (1930), follow by Dunham and Baldwin (1931), there has been limited progress in this area due to the complexity of directly observing and identifying interactions between bacteria and plants. The methods to assess competitiveness have traditionally been time-consuming and restricted to a small sample size. However, several techniques are now emerging, based on advances in imaging (Jeckel and Drescher, 2020) and sequencing technologies, which allow a high-throughput approach, as summarized in Table 2.

**TABLE 2:** The most frequently used methods to assess rhizobial competitiveness for nodulation.

**Method**	**Principle**	**Pros**	**Cons**	**Example of latest studies using this method to assess competitiveness**
Antibiotic markers Graham, 1969; Josey et al., 1979	Scoring rhizobial infection by plating nodule samples on suitable selected media	• No need for sophisticated equipment; • No need to genetically modify the strains; • Competitiveness of strain not affected.	• Limited number of strains can be evaluated; • Mixed nodules are often missed; • Relies on strain viability and culturability in different antibiotics which becomes very labor intensive.	Bogino et al., 2011; Laguerre et al., 2012; Bourion et al., 2018
Strain-specific fingerprints De Bruijn, 1992; Laguerre et al., 2003	Targeting specific plasmid profiles or genes; afterwards patterns of the resulting PCR products are analyzed	• Suitable as a first step to classify closely related strains in large collections.	• Requires strict standardization of reaction parameters; • Complex comparative analysis of banding patterns; • Does not allow the identification of mixed nodules.	Lardi et al., 2017; Irisarri et al., 2019; Pastor-Bueis et al., 2019
Sequential double staining to detect *gusA* and *celB* Sessitsch et al., 1996	Scoring of nodule infection by color detection after enzymatic reactions	• Allows efficient scoring of single or double nodule infections without requiring sophisticated equipment; • Stable insertion of marker genes, ideal for ecological experiments; • Can be used in large-scale assays and in the presence of an unmarked background population.	• Only possible to score two tagged strains simultaneously; • Toxic buffers are needed for the enzymatic reaction to distinguish nodule occupancy; • Not possible to recover viable rhizobia from stained nodules.	Sánchez-Cañizares and Palacios, 2013; Ferguson et al., 2020; [Bibr B185][Bibr B307]
Fluorescent proteins Stuurman et al., 2000	Detection of dual fluorescence by microscopy	• High resolution even at single cell level; • Viable rhizobia can be recovered from nodules.	• Only a few nodules per plant or a small plant sample size can be assessed due to microscopy complexity.	Checcucci et al., 2016; Regus et al., 2017; Bellabarba et al., 2020
NGS of full genome, core genes or accessory genes	Analysis of partial or full genome to identify individual strains	• Large numbers of rhizobial strains can be assessed simultaneously; • Measures relative strain diversity; • Tracks dynamic changes in strain populations.	• DNA samples are pooled, loosing information of individual strain-nodule relation; • Pre-sequencing of the genomes from the strains is needed; • Complex sequencing data analysis.	Ji et al., 2017; Burghardt et al., 2018; Boivin et al., 2020; Moeskjær et al., 2020
NGS of synthetic DNA fragments Mendoza-Suárez et al., 2020	Introduced unique barcodes (IDs) are detected by NGS to score bacterial populations in individual nodules	• Large numbers of rhizobial strains can be assessed simultaneously; • Strains not previously isolated and genome-sequenced can be identified; • Information at a nodule level; • Easy identification of mixed nodules; • Simultaneous assessment of competitiveness and effectiveness.	• High-throughput cloning methods are needed and bacteria library preparation; • A blue-light transilluminator is needed to detect GFP nodules from tagged strains vs native strains.	Mendoza-Suárez et al., 2020

**FIGURE 2:**
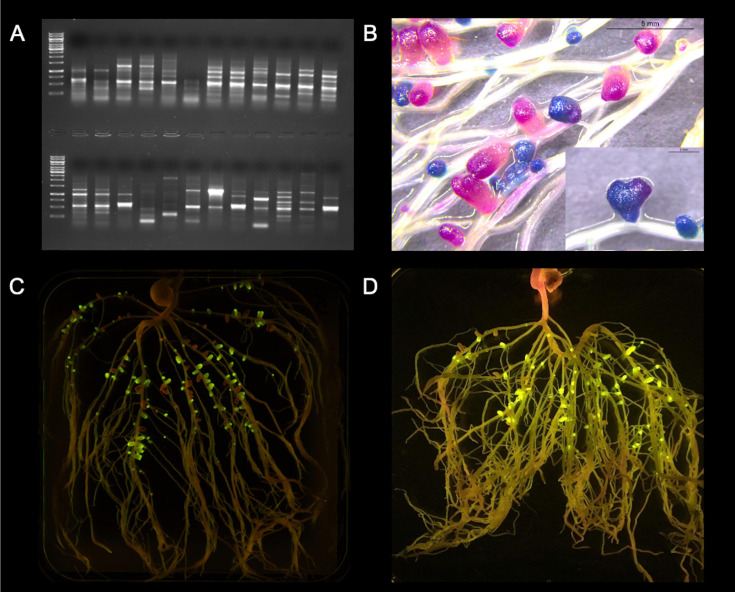
Visualization of some methods for assessing rhizobial competitiveness for nodulation. **(A)** Strain-specific genomic fingerprints: ERIC-PCR from same plant nodule isolates from a trapping assay using faba bean as a host; **(B)** Sequential double staining to detect *gusA* and *celB*: Pea roots were sequentially double-stained with Magenta-GlcA and X-Gal after thermal treatment, resulting in pink nodules formed by UPM791gusA (*gusA* constitutively expressed) and blue nodules formed by Rlv3841celB (*celB* constitutively expressed); **(C)** Fluorescent proteins: Rlv3841 labeled with mini-Tn7 J23104 GFP or mCherry, respectively; and **(D)** NGS of synthetic DNA fragments: Example of pea roots grown in non-sterile soil and exposed to a blue-light transilluminator. Tagged rhizobia, expressing GFP under Ps*nifH* control, lead to fluorescent nodules, while indigenous rhizobia do not. Photo credit: **(A,B,D)** Marcela Mendoza-Suárez; **(C)** Laura Clark.

Competition assays were initially performed using morphological, serological, or antibiotic markers that allowed for strain discrimination (Löhnis, 1930; Holland, 1966; Graham, 1969; Josey et al., 1979), followed by strain-specific genomic fingerprints (De Bruijn, 1992; Laguerre et al., 2003; Figure 2A). These methods are labor-intensive, as they involve isolating strains from nodules and plating them in selective media or complex and sometimes barely-reproducible PCR profile analyses. Marker genes such as *lacZ* (Drahos et al., 1986) and *luxAB* (O’Kane et al., 1988) facilitated observation of the bacteria-plant interaction, although the downsides were that ß-galactosidase activity from *lacZ* had a high background in both rhizobia and the plant host, and the luciferase assays with *luxAB* needed sophisticated equipment to be detected.

A tool that simplified these assays was the use of marker genes and chemical staining to detect *gusA* (Streit et al., 1992), and the simultaneous detection of *gusA* and *celB* (Sessitsch et al., 1996) by enzymatic activities rendering colored products (Figure 2B), or by fluorescent proteins (Stuurman et al., 2000; Figures 2C,D). However, these assays are still restricted to inoculum mixes of only two strains. More novel approaches today allow high-throughput assays by using either NGS of full genomes, core genes, or accessory genes, or NGS of synthetic DNA fragments (Mendoza-Suárez et al., 2020). It is worth noting the promoter driving the expression of the reporter genes. While constitutive promoters have been standard, Wilson et al. (1995b) and Sessitsch et al. (1996) used promoters that express only in symbiosis and nitrogen-fixing conditions and are a more specific alternative, such as the *nifH* promoter. A broader, more novel approach is the use of a universal *nifH* synthetic promoter, based on a consensus sequence adapted to different rhizobia by Mendoza-Suárez et al. (2020). These reporters can be either replicated in stable low-copy plasmids (Pini et al., 2017; Geddes et al., 2019a), through a quick conjugation step with the strains of interest, or can be integrated in the genome by stable mini-Tn7 vectors (Romero-Jiménez et al., 2015). The transformational breakthrough in Mendoza-Suárez et al. (2020) is the ability to simultaneously assess competitiveness and estimate rates of nitrogen fixation in individual nodules of pea plants. This is achieved thanks to the combination of a module for evaluating N_2_-effectiveness that included a consensus *nifH* (nitrogenase) promoter driving nodule-specific expression of green fluorescent protein as reporter gene, together with a second module consisting of a unique synthetic nucleotide sequence as a barcode strain identifier (ID; Figure 2D), allowing the screening of large libraries of bacterial strains.

**FIGURE 3:**
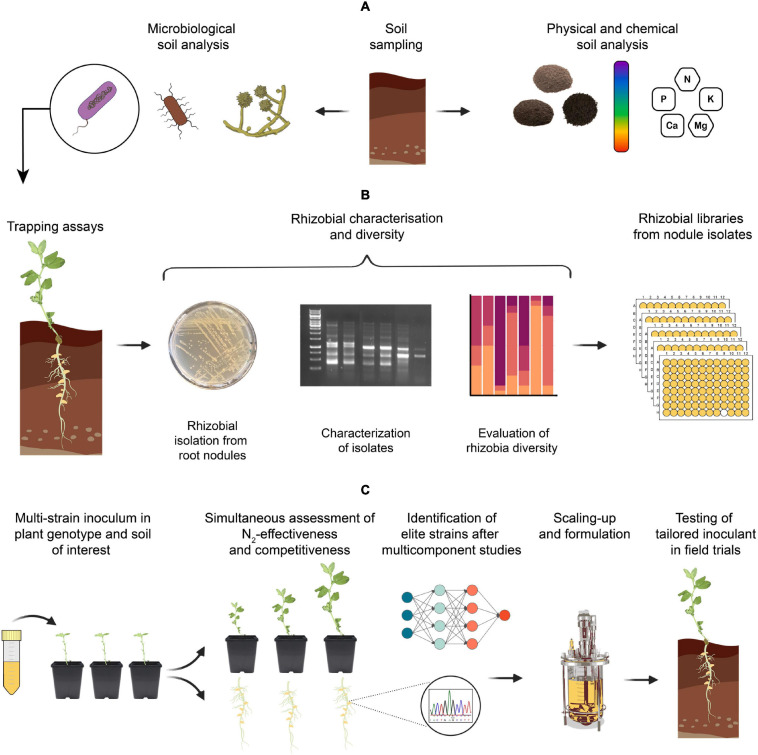
Suggested steps for developing elite rhizobial inoculants from native strains. **(A)** Perform physical, chemical, and microbiological soil analysis. Although we recognize the importance of full microbiome interactions with the plant, this review will focus on rhizobial inoculants. **(B)** Trapping assays using the desired host genotype and soil of interest, followed by the identification and characterisation of native rhizobia [i.e., Most probable number (MPN) – and strain-specific fingerprints], and preparation of rhizobial isolate libraries. **(C)** Multi-strain inoculations in the plant genotype using the same soil from which strains where isolated. Perform simultaneous assessment of rhizobial competitiveness and N_2_-effectiveness, followed by the identification of elite strains by next-generation sequencing (NGS). Analysis of multicomponent interaction studies using a multidisciplinary approach and final scaling-up and formulation based on the elite strains with best performance in the soil under study for a tailored inoculant.

We must not forget that competitiveness of a *Rhizobium* strain and how efficiently it fixes nitrogen is determined by its genetics and physiology, in interaction with the legume host genotype (Laguerre et al., 2003; Burghardt et al., 2018; Boivin et al., 2020) and the inoculation context, determined by soil influence (Batista et al., 2015) and climatic conditions (Frey and Blum, 1994; Vuong et al., 2017). The population of rhizobia in different soils is heterogeneous and varies quantitatively and qualitatively, responding to different abiotic and biotic factors (Graham, 2008; Kasper et al., 2019). A given elite strain may thrive in one climate or soil type but fail in a different environment to which it is poorly adapted. Therefore, when elite strains are found, these are for a particular soil and plant genotype, and may well not perform as well in a different plant genotype or different environment (Figure 3). It is of utmost importance that future competition assays are performed in non-sterile conditions; ultimately, rhizobial strains are not alone in the rhizosphere. It is therefore essential to apply and improve existing techniques, such as those of Burghardt et al. (2018) and Mendoza-Suárez et al. (2020), to other *Rhizobium* species. Like this, more studies could combine the assessment of a large number of strains for nodule occupancy using different host genotypes with the assessment of symbiotic N_2_-fixation in agricultural soils, whilst minimizing financial and time costs through increased testing efficiency.

## How Many Strains Are There in a Mixed Nodule?

Nodules containing more than one rhizobial strain, called “mixed nodules,” were identified several decades ago (Johnston and Beringer, 1976). Experimental results have shown that the percentage of mixed nodules is sufficiently high that it should be carefully considered in rhizobial studies, particularly in competition assays (May and Bohlool, 1983; Moawad and Schmidt, 1987). However, these competition experiments were traditionally conducted with two-strain mixtures. Thanks to the development of new methods to assess rhizobial competitiveness, advances in microscopy, and the continued decrease in price of NGS, it has now been possible to assess more simultaneously-competing strains, which has also made the easy identification of mixed nodules possible. These new studies suggest that not only are mixed nodules more common than thought previously, but they can also be occupied by more than two different strains. In fact, Mendoza-Suárez et al. (2020) found up to six different strains in a single nodule using unique barcode identifiers (ID). This also reinforces the hypothesis that there may be cooperative partnerships between strains.

## Cheaters and Plant Sanctioning Behaviors

Since multiple rhizobial strains may occupy the same nodule (Checcucci et al., 2016; Mendoza-Suárez et al., 2020), cheating behaviors have emerged (Sachs et al., 2010; Checcucci et al., 2016; Regus et al., 2017). Experiments with near-isogenic mutants demonstrated that a N_2_-ineffective mutant had a similar level of competitiveness to its parent (Amarger, 1981; Daubech et al., 2017), supporting the idea that plants do not select rhizobial strains by their fixing abilities at root entry level. Indeed, N_2_-ineffective rhizobial strains with superior competitiveness often gain advantage over N_2_-effective strains, despite offering suboptimal growth to their plant host (Sachs et al., 2010). Perennial legumes, where nodulation is a continuous and sequential process, establish symbiosis with less competitive rhizobial strains when they have no other choice. A study performed in *Caragana microphylla* with different *Mesorhizobium* strains demonstrated how the plant host could be infected with a more favorable rhizobial strain when the nodules were first occupied by less competitive rhizobial strains (Ji et al., 2017). However, if a legume had nodules occupied by its most favorable rhizobial strain first, it was unlikely that other rhizobial strains in the rhizosphere would be able to form new nodules on the roots, even if the density of the other strains was higher. This dynamism over time and life phase of the host plant has been reported in the nodule microbiome of *M. sativa*, indicating that its members strongly interact through cooperation and competition (Hansen et al., 2020). In fact, pea plants have been shown to tolerate intermediate fixers only when a better strain was not available (Westhoek et al., 2021). Post-infection plant control over bacteroid metabolism is essential due to the high fitness cost of nodule formation and bacteroid maintenance. The plant host must therefore monitor symbiotic performance and respond accordingly (Ledermann et al., 2021). However, plants cannot select effective nitrogen-fixing rhizobia from a mixture of effective and ineffective strains in the soil in the early stages of the symbiotic interaction (Westhoek et al., 2021). Instead, to avoid cheaters displacing effective symbionts once the infection has occurred, legumes limit cheating through host sanctions, which reduce the fitness of cheaters, and partner choice, where each partner can identify and reject forming relationships with cheaters (West et al., 2002; Kiers et al., 2003; Kiers and Denison, 2008; Sachs et al., 2010; Oono et al., 2011; Daubech et al., 2017; Westhoek et al., 2021). Sanctioning of ineffective nodules occurs by reducing the number of viable cells present in nodules and reducing their reproductive success (Kiers et al., 2003), by preferentially promoting nodule development in number and size with most BNF-efficient rhizobia due to the stimulation of plant cell multiplication and bacteroid differentiation (Laguerre et al., 2012), or presumably by reducing resource allocation to a nodule and shutting it down (Kiers et al., 2003; Westhoek et al., 2021). As seen in mixed nodules, where nodules are co-occupied by different strains, the host plant appears to spatially structure symbionts, separating in individual plant cells effective from ineffective partners. Therefore, when nodules are co-inhabited with a fixing strain, the inefficient strain is sanctioned rapidly in a cell-autonomous way (Regus et al., 2017). This means that plant sanctions are targeted specifically to those individual host cells housing ineffective partners, being an effective host strategy to sanction poorly performing strains. Even in mixed nodule infections, each individual plant cell appears to only have one strain. Plant nodules have been described as autonomous compartments where the host is able to actively rewire investment away from symbiont reproduction, and toward nitrogen fixation (Chomicki et al., 2020). In evolutionary terms, compartmentalization of host-microbe interactions helps to stabilize cooperation by allowing hosts to: (i) isolate symbionts and control their reproduction; (ii) reward cooperative symbionts and punish or stop interactions with non-cooperative symbionts; and (iii) reduce direct conflict among different symbiont strains in a single host (Chomicki et al., 2020). Hosts with symbiotic promiscuity (analyzed in detail in Perret et al., 2000) could face a stronger selection pressure to evolve effective post-infection discrimination, although the mechanisms by which the host monitors individual nodules, once formed, are still largely unknown (Chomicki et al., 2020).

## Current Production of Inoculants

The use of inoculants is well stablished in agricultural systems. Based on *Bradyrhizobium* strains, soybean is currently the crop that consumes the most inoculant production worldwide (Santos et al., 2019). Rhizobial strains have traditionally been isolated either from bulk soil (Figure 3A) in a semi-selective medium or with trapping assays designed for a soil and a host of interest (Figure 3B; Laguerre et al., 2003). For a rhizobial strain to be considered as a potential inoculant, the easiest approach is to first characterize the isolate in the laboratory and show its effectiveness at nitrogen fixation, its ability to successfully fight for rhizosphere colonization, and its relative competitiveness against native rhizobia for nodule occupancy. Screening for the relative ability of rhizobia to compete for the rhizosphere appears a quite good predictor of nodule occupancy as indicated by the strikingly linear impact that access to the rhizosphere has been shown to have on nodule occupancy (Salas et al., 2017). Once these characteristics have been determined, it is crucial to test rhizobial strains in greenhouse and field trials to analyze the effects of the inoculant treatment on agronomic traits, such as: nodulation, plant biomass, shoot N content, grain yield, and/or grain N content (Werner and Newton, 2005; Lupwayi et al., 2006). Once the screen for a set of elite rhizobia performing well in the rhizosphere has been made, these wild type strains can then be incorporated into more complex assays, such as Tn-Seq/INSeq or BarSeq, where the mutagenesis can be done in soil conditions where a natural microbiome will be present, or genome comparisons between an elite strain versus non-competitive strains to identify the genetic determinants involved in competition.

Those strains selected as inoculants are typically grown at a fermenter scale and used to coat seeds of compatible legume hosts to introduce them into the soil (Figure 3C; diCenzo et al., 2019). However, this inoculation method often results in a high density of bacteria near the seed, with nodulation restricted to the upper tap root but reduced in the more distal part of the tap root and the lateral roots due to the low density of the inoculant strain in the bulk soil (Vlassak and Vanderleyden, 1997). Alternatively, in-furrow inoculation – placing the rhizobial strain in the seed bed – is an approach that enhances rhizobial motility and consequently, increases nodule occupancy (López-García et al., 2009). It also works well for legumes infected by crack entry, such as peanut and bradyrhizobia, as the chances of encountering breaks in the secondary root epidermis and establishing symbiosis are increased (Bogino et al., 2011). Therefore, to maximize rhizobial inoculant efficacy, research must focus on the following key aspects: the intrinsic characteristics of the rhizobial strain; the delivery system into the soil, known as inoculant formulation; the optimization of the production process by industrial fermentation, and the compatibility with the farmer’s practices (Catroux et al., 2001; Temprano et al., 2002; Herridge et al., 2008; Checcucci et al., 2017; Pastor-Bueis et al., 2019).

Although an elite strain is an essential prerequisite in the development of successful inoculants, there are several factors to consider when working in laboratory conditions. Firstly, nodule-dominant strain genotypes from soil populations do not necessarily show superior competitiveness for nodulation compared to minor occupants when evaluated under non-soil conditions (Laguerre et al., 2003). Secondly, legumes grown in sterile conditions under nitrogen starvation regimes can show significant growth differences between the inoculated and non-inoculated treatments. However, real agricultural conditions are never entirely devoid of nitrogen, and populations of native rhizobia are usually present (Wilson et al., 1995a). As the sole inoculant applied to legumes under laboratory or controlled conditions, specific strains of rhizobia may increase nitrogen fixation (Wielbo et al., 2010); however, once these strains are applied to legumes under real agricultural conditions, they often fail in the competition with native strains for nodulation due to soil conditions, rooting depth, humidity, temperature, and the inoculant formulation (López-García et al., 2002; Naeem et al., 2004; Yates et al., 2011; Drew et al., 2012). Finally, another important trait in an inoculant is the survival rate; both in the carrier – a wide range of carriers can be used (peat, compost, vermiculite, perlite, and sand) – and in the soil. The strain used as the inoculant needs to be either robust or well protected in order to survive under harsh conditions (Parnell et al., 2016). Rhizobia should survive and grow in the soil in the absence of the host plant and be able to colonize the host plant rhizosphere prior to competition for root infection and nodule formation (Laguerre et al., 2003). The use of coated seeds is the most convenient delivery system, but while rhizobia survive well in inoculant formulations, some die rapidly after seed-coating owing to osmotic and desiccation stress (O’Callaghan, 2016; Atieno et al., 2018). Formulation of inoculants is a crucial issue, but little research has been conducted on this subject. It can improve field performance, shelf life, and stability while reducing variability (Parnell et al., 2016; Santos et al., 2019). Both liquid and solid formulations are widely used. Currently, the most widespread dry formulation consists of peat as a carrier, plus other additives such as bacterial protectors and adhesives (Bashan et al., 2014; Atieno et al., 2018; Santos et al., 2019). Drying of microorganisms has been recognized as an efficient way of long-term preserving; however, desiccation is a physiologically challenging process, so protectants are added externally to the bacterial cells prior to drying (Berninger et al., 2017). Among other mineral or organic carriers, compost and biochar—the solid, carbon-rich product of heating biomass with the exclusion of air, with high porosity, large specific surface area, adsorption ability, and high cation exchange capacity—have been proposed as carriers with outstanding properties (Albareda et al., 2008; Arif et al., 2017; Egamberdieva et al., 2017; Song et al., 2020). It has also been shown that the combination of an inoculant strain together with a carbon source, such as glycerol in microgranules, confers a competitive advantage to the inoculant bacterium (Duquenne et al., 1999; Sessitsch et al., 2002). Biomaterials adopted from the field of drug delivery have been proposed as a technological opportunity for developing an advanced seed-coating (Zvinavashe et al., 2019). These authors have developed a seed-coating based on silk fibronin and trehalose that stabilizes and preserves rhizobia in saline and, possibly, arid environments. However, the non-biological components of the formulations remain key bottlenecks in the commercial development of inoculants (Bashan et al., 2014; Pastor-Bueis et al., 2019). For all these reasons, experiments in soil conditions are crucial, with the ultimate goal being the design of a successful inoculant based on an elite native strain with an adequate formulation which can result in greater grain yields than without the use of rhizobial inoculants. Or, in some cases, grain yields can even be similar or higher compared to chemical fertilization practices in inoculated fields with native strains (Mulas et al., 2015). Of course, the latest results have to be considered in an arid soil context, where the loss of total nitrogen input is leached at rates of up to 58% (Hu et al., 2010). In seeking elite strains for several pulse legumes in areas of difficult soils placing substantial stress on inoculant survival, Howieson et al., 2000, successfully developed an inoculant program based on cross-row experiments in successive years. The authors screened for nitrogen fixation, edaphic adaptation and performance *in situ* of rhizobial strains originally collected from the Mediterranean region, which reflects the edaphic characteristics of the target soils in Southern Australia. Inoculation with these better adapted strains, selected for their superior N_2_-effectiveness, increased yield and nodulation in legume crops in infertile soils combining acidity and desiccation. Moreover, despite the competition for nodulation by background rhizobia at the site, assessment of nodule occupancy by the inoculant strains revealed all were present in >90% of nodules, securing the establishment of pulse crops in difficult soils. Below we explain why rhizobial inoculant strains that are applied to legumes in field conditions often fail to nodulate legumes in competition with native strains (López-García et al., 2002; Naeem et al., 2004; Yates et al., 2011; Drew et al., 2012).

## Genetic Stability of Inoculant Strains

The genetic instability of inoculant strains and the exchange of symbiotic plasmids contribute to the diversity of naturalized populations and the lack of inoculant persistence (Ronson and Lowther, 1995). Several reports based on the phylogeny of *nod* genes—located in the symbiotic plasmid or symbiotic islands—have shown that the Sym plasmid is not strictly associated with the chromosomal background in natural populations of rhizobia (Schofield et al., 1987; Young and Wexler, 1988; Laguerre et al., 1992; Louvrier et al., 1996; Andrews et al., 2018). Since lateral gene and plasmid transfers are the major drivers of symbiotic phenotype evolution, bacterial genospecies are not ecologically relevant for symbiotic traits (Kumar et al., 2015; Andrews et al., 2018; Boivin et al., 2020). Symbiovars reflect the symbiosis plasmid rather than chromosome diversity (Kumar et al., 2015).

While the transfer of symbiosis genes to bacteria adapted to local soil conditions can allow them to become symbionts of previously incompatible legumes growing in those soils (Sullivan et al., 1995; Barcellos et al., 2007; Rivas et al., 2007; Andrews et al., 2018), in the case of inoculant strains this common and widespread phenomenon in fact results in the opposite outcome. Natural transfer of symbiotic islands by mobile integrative and conjugative elements has been demonstrated in field trials with mesorhizobia (Sullivan et al., 1995; Sullivan and Ronson, 1998; Nandasena et al., 2006, 2007; Hill et al., 2021), where resident non-nodulating bacteria accepted symbiotic genes from the inoculant *Mesorhizobium* strain, often resulting in highly competitive strains with poor nitrogen fixation capabilities that outcompete the original inoculant, potentially rendering it ineffective (Sullivan et al., 1995; Nandasena et al., 2007; Sotelo et al., 2011). Self-transmissible plasmids may be maintained within field isolates because they confer selective advantages on host strains (Meade et al., 1985). Genes required for the catabolism of plant exudates or the utilization of a range of carbon sources are often located on plasmids. For example, the genes involved in the degradation of rhamnose were reported to be induced by root extracts from the host plant, playing a role in competition of *R. leguminosarum* bv. *trifolii* in the early stages of the symbiotic interaction (Oresnik et al., 1998). Mutants unable to utilize this carbon source had impaired competitive abilities. The same applies to homoserine, an amino acid abundantly exuded by pea roots. *R. leguminosarum* bv. *viciae* strains able to use homoserine as a carbon and nitrogen source were found to be prevalent in pea nodules (Hynes and O’Connell, 1990; Vanderlinde et al., 2014). However, this does not exclude the possibility that some genes present on the chromosome may also contribute to the symbiotic phenotype and its variation (Boivin et al., 2020; Ferguson et al., 2020).

## Erratic Performance of Inoculants

In many locations, native rhizobial populations are either not effective, or do not occur in sufficient number to meet the nitrogen demand of promiscuous cultivars, leading to a safer inoculation approach with exotic elite rhizobial strains instead of relying on resident strains of unknown potential (Chibeba et al., 2017). Moreover, although rhizobia are ubiquitous in the soil, the introduction of new plant species in a different location usually results in a lack of co-evolved rhizobial strains in soils abroad (Mpepereki et al., 2000; Giller, 2001; Simonsen et al., 2017; Bouznif et al., 2019). Successful introduction of crops into new regions is, therefore, dependent on inoculation with exotic rhizobia. Many examples in the Southern hemisphere illustrate this phenomenon, such as African soils and soybean cultivars (Okogun and Sanginga, 2003; Abaidoo et al., 2007; Klogo et al., 2015), forage and grain legumes in Australian and New Zealand soils (Ronson and Lowther, 1995; Hill et al., 2021), soybean in Argentina (Iturralde et al., 2019), common bean in Brazilian soils (Hungria et al., 2003), or forage production of *Lotus corniculatus* and clover in Uruguay (Sotelo et al., 2011, Irisarri et al., 2019). However, this leads to another problem with promiscuous cultivars: commercial inoculants based on exotic rhizobial strains selected for their efficiency in nitrogen fixation fail to establish a successful symbiosis by not being competitive against inefficient native rhizobia (Martínez-Romero, 2003; Pastor-Bueis et al., 2019; Shamseldin and Velázquez, 2020). Indeed, the absence of the inoculated strain in the nodules leads to low productivity, attributed to poor performance of the inoculant, and lack of consistency in field performance (Sotelo et al., 2011; Irisarri et al., 2019). The infection of legume crops by native populations of ineffective but competitive local rhizobia is known as “the competition problem” and causes yield decreases of legume crops (Triplett and Sadowsky, 1992; Friesen, 2012). Due to these inoculation problems, farmers prefer to use chemical fertilizers rather than rely on the capacity of the inoculants to carry out an efficient BNF (Pastor-Bueis et al., 2019). Therefore, the design of tailored inoculants by selecting naturally-evolved and locally-sourced rhizobia with outstanding symbiotic performance (effectiveness) that are already adapted to the agroclimatic conditions of a particular region (competitiveness) is of fundamental importance for agricultural lands worldwide in order to tackle this competition problem and increase crop yields without the use of nitrogen fertilizers (Chibeba et al., 2017; Pastor-Bueis et al., 2019). Indeed, local adaptation is a more important evolutionary force shaping microbial cooperation than is partner choice (Batstone et al., 2020). External factors are major determinants of the high competitiveness of soil native populations, such as the physiological condition and the growth age of the rhizobial culture, the distribution of the bacterial cells into the soil, the presence of salt and the soil pH of the sampling location, the soil surface coverage and soil temperatures during the crop season, or the resistance to the herbicides applied to the soil as part of the agricultural practice (López-García et al., 2002; Albareda et al., 2006; Iturralde et al., 2019). Therefore, different field scenarios need to be investigated. Methods to conduct field trials with those elite strains are being developed to ascertain their superiority in fixing nitrogen in the presence of native and/or commercial strains (Mendoza-Suárez et al., 2020).

## Moving Toward Tailored Inoculants

The addition of chemical fertilizers has a negative impact on the soil microbiome, as plants no longer need to interact with beneficial bacteria to access the nutrients that are being externally supplied and, therefore, the diversity of the microbial community in the root environment is reduced (Zhu et al., 2016; Kavamura et al., 2018). A study performed in wheat has shown that chemical fertilizers reduce the number of bacteria associated with the roots that solubilise nutrients such as nitrogen, potassium, phosphorous, iron, and zinc (Reid et al., 2021). Strikingly, the number of growth-promoting bacteria living on the roots fell from 91% of total bacteria for unfertilized plants, to just 19% for those that received the fertilizer dose. Chemical nitrogen inputs for a period of 22 years have guided the evolution of less-mutualistic rhizobia which produce 17–30% less biomass on *Trifolium* species compared to plants inoculated with rhizobia from non-fertilized control plots (Weese et al., 2015), which clearly shows the great negative environmental consequences of long-term input of chemical fertilizers in agricultural systems. Therefore, optimizing microbiome function is essential for sustainable agriculture (Hartmann et al., 2015).

On the other hand, Batstone et al. (2020) have found that after five plant generations, *M. truncatula* select for a more efficient *E. meliloti* symbiont. The superior host benefits were observed when the plant-microbe partnership shared an evolutionary history. Incorporating rhizobial strains with closely-related hosts is more likely to be effective, but it is necessary to give the new microbe sufficient time to adapt to their new environment. Schlaeppi and Bulgarelli (2015) were the first authors to foresee next-generation agriculture that aims to customize practices and tools, such as microbial inoculants, inspired by the concept of personalized diagnosis in medicine. Bell et al. (2019) proposed a customizable field-scale microbial inoculant that could have long-lasting effects with appropriate implementation. These innovative biofertilizer technologies based on tailored design and the implementation of effective agricultural microbiome manipulations and management strategies will benefit both consumers and producers of worldwide food supply (Busby et al., 2017; Mitter et al., 2021). Particularly in marginal soils of arid and semi-arid regions, inoculation is a crucial agricultural practice, with BNF being the major way to introduce nitrogen (Zahran, 1999). Not only that, but rhizobial inoculants also have other important and promising ecological applications, such as the restoration of habitats or the conservation of endangered plant species (Rodríguez-Echeverría et al., 2003; Thrall et al., 2005; Beyhaut et al., 2014; Navarro et al., 2014).

Although, as we have seen, it is well known that taking into account *Rhizobium* genotype (Gr) interactions together with root and soil microbiota ecology (M), plant host genotype (Gp), and/or environment (E), is important for evaluating whether selected strains are going to be suitable as inoculants (Figure 3C; Roskothen, 1989; Hungria and Vargas, 2000; Sessitsch et al., 2002; Busby et al., 2017; diCenzo et al., 2019)—note that authors have used different abbreviations—much research on competition has nevertheless focussed on changing only one or a few variables at a time. This is due to the complexity of distinguishing specific strains of interest, the limitations of running scaled-up plant experiments, and a lack of knowledge in understanding the ecological component of heterogenous communities and complex environments (including field trials). Of course, the big challenge is which one of these variables is most important to study or control? Although multicomponent interaction studies can be perceived as an overwhelming and titanic task, we can rely on a multidisciplinary approach to tackle the problem. Indeed, more and more multicomponent interaction studies are being conducted (Vuong et al., 2017; Burghardt et al., 2018; Gunnabo et al., 2019; Batstone et al., 2020; Mendoza-Suárez et al., 2020; Fagorzi et al., 2021), and they are already generating important knowledge to help understand some of these interactions and give more weight to the performance of rhizobial inoculants. Additionally, the rapid progress in NGS with open-source laboratory equipment automation (Figure 3C; Wong et al., 2018; Faiña et al., 2020), and the application of machine learning in big data analysis of microbiome studies (Cammarota et al., 2020; Ghannam and Techtmann, 2021) and in biological-image analysis (Berg et al., 2019; Chung et al., 2020; Tausen et al., 2020), will make multicomponent interactions studies more achievable, providing the opportunity to identify a larger number of elite strains in less time. The introduction of a wider number of variables in the experimental assays conducted to identify elite strains will increase the probability of designing rhizobial inoculants with better performance under field conditions. Once elite strains are identified in greenhouse experiments exploring multicomponent interactions, strict testing needs to be conducted in field trials before their commercialisation (Soumare et al., 2020; Figure 3C). Due to the complexity and genetic diversity within the soil and plant microbiome, it is unlikely that one formulation will be effective for all fields (Mitter et al., 2021). Yet, it might be unrealistic to design tailored inoculants for each individual field (Parnell et al., 2016). However, it may be possible to first carry out broader and cheaper assays to assess soil factors such as pH, nutrient and organic material content, and then correlate the physicochemical properties of the field with an already established collection of strains that perform well in those field conditions. Tailored inoculants may also become cheaper with increased production, which gives an opportunity to learn how to improve the production process and begin a positive-feedback cycle of increasing demand and falling prices (Schmidt et al., 2017; Lafond et al., 2020). For this reason, we suggest that efforts should be made to develop a multidisciplinary validation system that targets optimal performance for a given set of components. Thanks to progress in multicomponent interactions and multidisciplinary studies, it could become possible to offer farmers tailored rhizobial inoculants in the near future by conducting full physicochemical and microbiological soil analysis, in a similar way to fertilizer consultants offering an “inoculant recommendation” for specific legume crops to grow in the next agricultural cycle (Figure 3). This inoculant advice would work in the same way that chemical fertilizer recommendations based on soil testing are currently offered and would reinforce and complement the farmer’s current agricultural practice.

## Conclusion

Ensuring the sustainability of agriculture becomes more important in light of future challenges such as climate change or the rapid growth of the human population (Schlaeppi and Bulgarelli, 2015). Next-generation agriculture will greatly benefit from the development of rhizobial bioinoculants based on elite strains that combine effectiveness and competitiveness under field conditions. The aforementioned areas of research in competition are shedding light on many of the processes that affect the performance of the inoculant and should be taken into account. The advances in imaging and sequencing technologies are improving our knowledge about all the stages in the symbiotic process, deciphering the mechanisms of cheating and cooperative bacterial behaviors, uncovering the genetic features behind competition and allowing high-throughput approaches to assess this trait, also under field conditions. Moreover, machine learning will allow us to automate assays and data analysis. In the longer term, these advances will contribute to solving the competition problem by allowing cost-efficient design and production of site-specific inoculants.

## Author Contributions

MM-S and CS-C wrote the manuscript. SA and PP provided critical feedback. All authors conceived and approved the final version of the manuscript.

## Conflict of Interest

The authors declare that the research was conducted in the absence of any commercial or financial relationships that could be construed as a potential conflict of interest.

## Publisher’s Note

All claims expressed in this article are solely those of the authors and do not necessarily represent those of their affiliated organizations, or those of the publisher, the editors and the reviewers. Any product that may be evaluated in this article, or claim that may be made by its manufacturer, is not guaranteed or endorsed by the publisher.
